# Involvement of adenosine signaling pathway in migraine pathophysiology: a systematic review of preclinical studies

**DOI:** 10.1186/s10194-022-01412-0

**Published:** 2022-04-05

**Authors:** Janu Thuraiaiyah, Lili Kokoti, Mohammad Al-Mahdi Al-Karagholi, Messoud Ashina

**Affiliations:** 1 Danish Headache Center, Department of Neurology, Faculty of Health and Medical Sciences, Rigshospitalet – Glostrup, University of Copenhagen, Valdemar Hansen Vej 5, 2600 Glostrup, Denmark; 2 Danish Headache Knowledge Center, Rigshospitalet – Glostrup, Valdemar Hansens Vej 5, 2600 Glostrup, Denmark

**Keywords:** Headache, Adenosine receptor, Pre-clinical, Pain

## Abstract

**Background:**

Adenosine is a purinergic signaling molecule with a wide range of physiological functions including anti- and pronociceptive properties. Adenosine receptors are expressed in the trigeminovascular system, and adenosine receptor antagonist, caffeine, relieves migraine headache. We performed a systematic review of the literature of preclinical data addressing the role of adenosine in migraine pathophysiology.

**Methods:**

PubMed and EMBASE were searched for pre-clinical studies on the role of adenosine in migraine pathophysiology on September 5^th^, 2021.

**Results:**

A total of 2510 studies were screened by title and abstract. Of these, thirteen pre-clinical studies evaluating adenosine, adenosine A1, A2A and A3 receptors were included.

These studies showed that adenosine signaling pathway is involved in controlling vascular tone. Furthermore, electrical stimulation of the trigeminal ganglion modulates the expression of adenosine A_1_ and A_2A_ receptors in the trigeminal ganglion and trigeminal nucleus caudalis implicating adenosine signaling pathway in pain transmission.

**Conclusion:**

Preclinical studies showed that adenosine has a dual effect on vasodilation and trigeminal pain pathway due to different receptor activation, suggesting a possible role of adenosine in migraine pathophysiology. Studies investigating pharmacological characteristics of subtypes of adenosine receptors are needed to further elucidate their role as a potential target for migraine treatment.

## Introduction

**Fig. 1:**
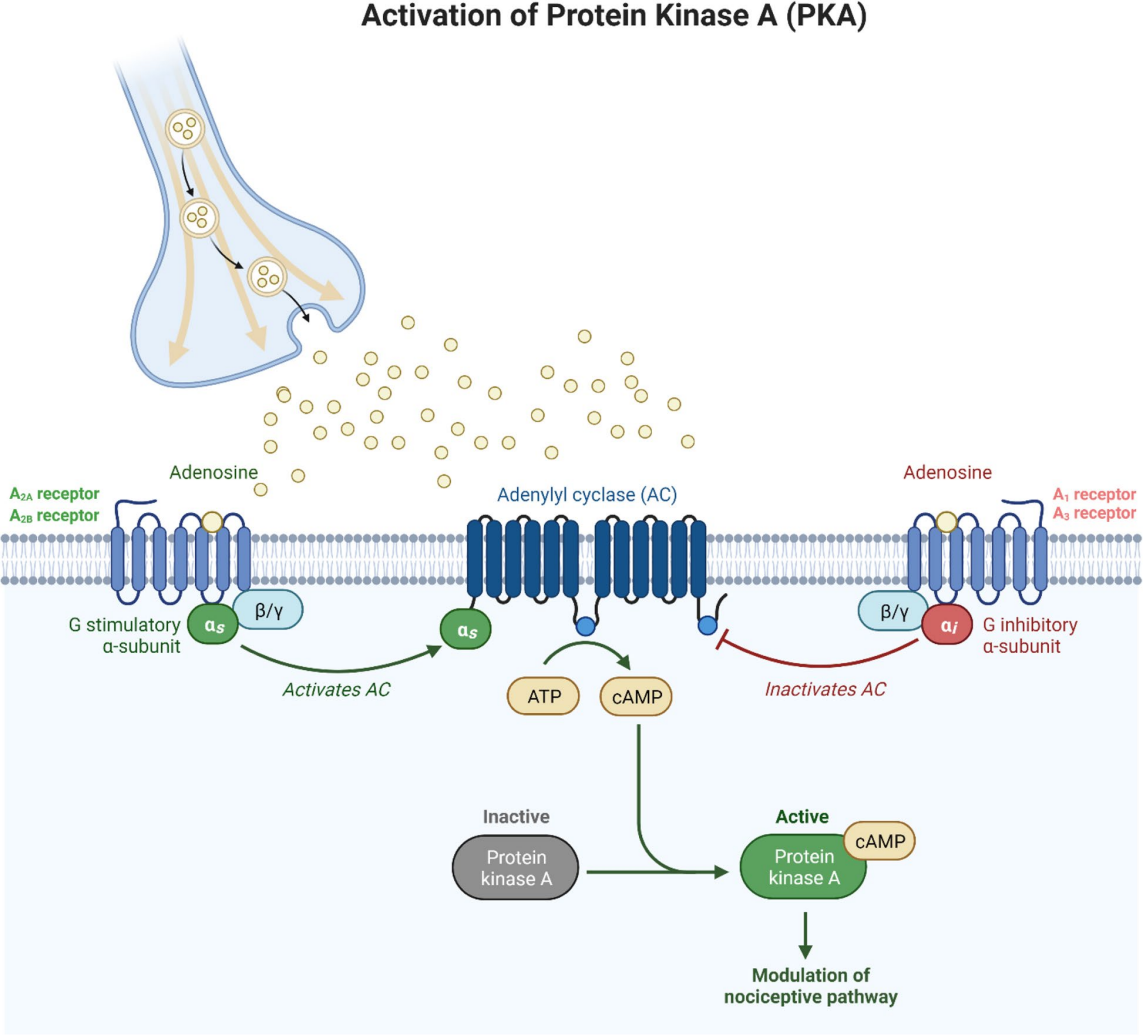
Adenosine signaling pathway. Adenosine binds to G-protein coupled receptors (GPCR) resulting in either activation (adenosine A_2A_ and A_2B_ receptors) or inactivation (adenosine A_1_ and A_3_ receptors) of adenylyl cyclase (AC). Activation of AC increases the formation of cyclic adenosine monophosphate (cAMP) which binds to protein kinase A (PKA). Active PKA then phosphorylates and thereby modulates cellular responses. ATP: adenosine triphosphate

Adenosine, a vasoactive amine produced by the hydrolysis of adenosine monophosphate (AMP) or S-adenosylhomocysteine (SAH) [43], is involved in numerous physiological processes such as metabolism, inflammation, respiration and pain [39]. Adenosine binds to four G-protein coupled receptors (GPCR), A_1_, A_2A_, A_2B_ and A_3,_ with a unique profile of tissue distribution, signaling pathways and function (Table 1 & Fig. 1) [20, 21]. Adenosine receptors activate the mitogen-activated protein kinase (MAPK), leading to survival, cell growth, and differentiation [20], and modulate the activity of adenylate cyclase [11, 20], the enzyme that regulates intracellular concentration of cyclic adenosine monophosphate (cAMP) [38]. Adenosine A_1_ and A_3_ receptors are coupled to a Gα_i_ subunit that downregulates cAMP by inhibiting adenylate cyclase [20, 38], while A_2A_ and A_2B_ receptors are coupled to a Gα_s_ subunit which stimulates adenylate cyclase to upregulate cAMP [20, 38]. A_1_ and A_3_ receptors are considered to have anti-nociceptive effects, whereas, activation of A_2A_ and A_2B_ receptors induces nociception [11]. Adenosine A_1_ receptors are expressed at the trigeminovascular system (TVS), including the trigeminal ganglion (TG) and trigeminal nucleus caudalis (TNC) [6, 32], which is considered to be the anatomical and physiological substrate of migraine pain [2]. Stimulation of this receptor causes inhibition of the TVS by reducing neuronal firing from the trigeminal nucleus and decreasing the release of calcitonin gene-related peptide (CGRP) [6, 13]. A_2A_ and A_2B_ receptors are located in vascular smooth muscle cells [20, 38] and in pre- and postsynaptic nerve terminals [38], and stimulation of these receptors causes dural vasodilation [16], leading to stimulation of the TVS. Collectively, adenosine signaling pathways are complex and might be involved in headache and migraine pathophysiology.

**Table 1:** Adenosine receptors and their functions

Receptor	Subunit	Signaling pathway
Adenosine A_1_ receptor	Gα_i_-subunit	Inhibits adenylate cyclase, decreases cAMP formation [20, 38], leading to activation of K_ATP_ channels [4, 26, 35, 39] and inactivation of BK_Ca_ channels [28]
	G_βγ_ subunits	Stimulates PLC and increases IP_3_ [20, 38]
Adenosine A_2A_ receptor	Gα_s_-subunit	Activates adenylate cyclase and increases cAMP formation [20, 38], leading to activation of K_ATP_ channels [27]
	Pertussis toxin insensitive Gα15 and Gα16 proteins	Activates PLC and upregulates IP_3_ [20]
Adenosine A_2B_ receptor	Gα_s_-subunit	Stimulates adenylate cyclase, increases cAMP formation [38], leading to activation of K_ATP_ channels [27]
	G_q_ subunit	Activates PLC and upregulates IP_3_ [38]
Adenosine A_3_ receptor	Gα_i_ -subunit	Inhibits adenylate cyclase and decreases cAMP [11, 20]
	G_βγ_ subunits	Increases the activity of PLC and PLD [38]
All		Modulates MAPK [20]

*cAMP* cyclic adenosine monophosphate, *IP*_*3*_ inositol 1,4,5-triphosphate, *MAPK* mitogen-activated protein kinase, *PLC* phospholipase C, *PLD* phospholipase D

Here, we systematically review preclinical studies on the involvement of adenosine in trigeminal pain pathway, to make the case that adenosine signaling pathways may play a role in migraine and discuss adenosine receptors as potential target for future treatment of migraine (Table 2).

**Table 2:** Molecules presented here that target adenosine receptors. None are approved for treatment

Name	Target	Clinical use
**Adenosine receptor agonists**		
GR79236	Highly potent and selective adenosine A_1_ receptor agonist [12]	Ischemic heart disease [3, 25], sleep apnea [5], modulation of lipolysis and insulin sensitivity [18]
GR79236X	Selective adenosine A_1_ receptor agonist [6]	Ischemic heart disease [44] and dental pain [41]
GR190178	Low efficacy (partial) adenosine A_1_ receptor agonist [13]	Cluster headache [13]
CGS21680	Selective adenosine A_2A_ receptor agonist [6]	Huntington’s disease [7], spinal cord injury [36], bone regeneration [33] and overactive bladder [47]
2-CI-IB-MECA	Selective adenosine A_3_ receptor agonist [6]	Atrial function [49], damage of the optic nerve and white matter ischemic damage [14]
**Adenosine receptor antagonists**		
Caffeine	Non-selective adenosine A_1_ and A_2A_ antagonist [34]	Treatment of headache, pain, apnea in premature children and neurodegenerative diseases [10]
DPCPX	Selective adenosine A_1_ receptor antagonist [6, 13]	Depression [42], cancer [8, 30, 50] and neuroprotection [37]
SCH58261	Potent and highly selective A_2A_ receptor antagonist [15]	Spinal cord injury [36], epilepsy [29] and preeclampsia [40]
JNJ-41942914, JNJ-39928122, JNJ-40529749, JNJ-40064440, and JNJ-41501798	A_2A_ receptor antagonists [16]	None

## Method

### Data source

We conducted two searches on PubMed and Embase on September 5^th^, 2021. Firstly, we searched “(“adenosine”[MeSH Terms] OR “adenosine”[All Fields] OR “adenosin”[All Fields] OR “adenosine s”[All Fields] OR “adenosines”[All Fields]) AND (“migrain”[All Fields] OR “migraine disorders”[MeSH Terms] OR (“migraine”[All Fields] AND “disorders”[All Fields]) OR “migraine disorders”[All Fields] OR “migraine”[All Fields] OR “migraines”[All Fields] OR “migraine s”[All Fields] OR “migraineous”[All Fields] OR “migrainers”[All Fields] OR “migrainous”[All Fields])”. Secondly, we searched for “(“adenosine”[MeSH Terms] OR “adenosine”[All Fields] OR “adenosin”[All Fields] OR “adenosine s”[All Fields] OR “adenosines”[All Fields]) AND (“headache”[MeSH Terms] OR “headache”[All Fields] OR “headaches”[All Fields] OR “headache s”[All Fields])”. Both searches were restricted to English language.

### Selection criteria and study inclusion

Studies were restricted to pre-clinical or clinical studies that investigated adenosine, adenosine agonist, adenosine antagonist, adenosine deaminase, adenosine deaminase inhibitor and adenosine reuptake inhibitors in headache and migraine pathophysiology. We excluded reviews, meta-analysis, conference proceedings and case reports.

Two investigators (J.T. and L.K.) screened all studies by title and abstract, followed by full text screening to confirm eligibility. References of the included studies were screened to find studies that were missed by the search. For each included study, both investigators (J.T. and L.K.) extracted hypothesis or purpose of the study, method, sample size, main outcome, conclusion, and limitations. Any disagreements were resolved through discussion by the two investigators (J.T. and L.K.). If the conflict remained, a third investigator (M.M.K) made the final decision.

## Results

**Fig. 2:**
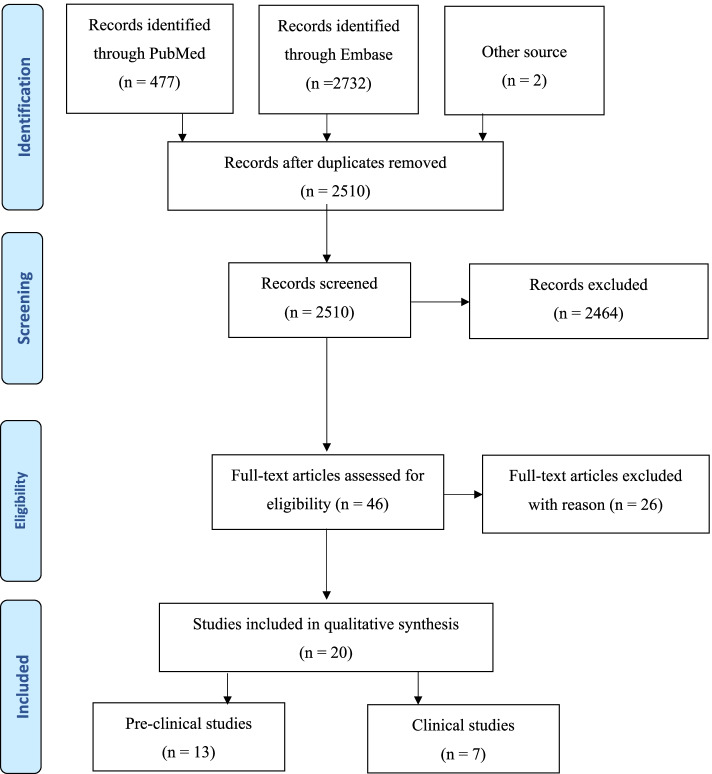
Flow chart of search strategy

The database search identified 3209 citations of which 701 were duplicates (Fig. 2). An additional two studies were included through a manual search of identified primary articles. A total of 2510 studies ﻿were screened by title and abstract and 44 were full text screened. Of these, 20 studies were included – 13 preclinical (Table 3) and 7 clinical studies. Data for clinical studies has been published recently [45].

**Table 3:** Summary of preclinical studies

Author	Purpose of the study	Study population (n)	Method	Main outcome(s)	Conclusion
Arulmani et al. [1]	To investigate the effects of GR79236 on capsaicin-induced carotid hemodynamic changes and plasma CGRP release	Pigs (15)	Capsaicin infusion after treatment with adenosine A_1_ receptor agonist, GR79236, or vehicle (saline)	GR79236 attenuated capsaicin-induced carotid hemodynamic changes dose-dependently, but not CGRP release	GR79236 might have potential as migraine treatment because of its vasoconstrictive effects rather than its inhibition of trigeminal CGRP release
Carruthers et al. [6]	To investigate the mechanisms of CGRP release and its potential modulation by adenosine agonists	Rats (*n* = NR)	**1)** Immunocytochemistry and western blotting **2)** In vitro application of forskolin and adenosine A_1_ receptor agonist (GR79236X), adenosine A_1_ receptor antagonist (DPCPX), adenosine A_2A_ agonist (CGS21680) or A_3_ receptor agonist (2-CI-IB-MECA) to trigeminal ganglion neuron cultures. CGRP was measured using ELISA	**1)** Adenosine A_1_ receptor was expressed in the trigeminal ganglion **2)** GR79236X inhibited forskolin stimulated CGRP release **3)** DPCPX abolished inhibition of CGRP release mediated by GR79236X **4)** CGS21680 and 2-CI-IB-MECA did not have effect on forskolin-induced CGRP secretion	A_1_ adenosine receptors on CGRP-positive neurons can inhibit cAMP induced CGRP release from trigeminal neurons. Adenosine receptors has significant potential for development as therapeutic targets for pain
Faraci et al. [9]	To investigate the vascular responses of the dura mater to adenosine	Dogs (*n* = 9)^a^	Intravenous infusion of 5 µM/kg/min adenosine while measuring blood flow with labelled, radioactive microspheres	Adenosine infusion showed **1)** aortic pressure decreased **2)** vascular resistance in the dura decreased **3)** blood flow to the dura increased **4)** no effect on cerebral blood flow	Adenosine did not affect cerebral blood flow but increased dural blood flow Vasomotor responses of the dural circulation might contribute to some forms of vascular headache
Goadsby et al. [13]	To investigate the role of adenosine A_1_ receptors in an animal model of nociceptive activation of the trigeminovascular system	Cats (*n* = 14)	Blood samples were taken at: baseline, after electrical stimulation of superior sagittal sinus (SSS), after electrical stimulation and GR79236 or GR190178. A single dose of DPCPX was administered after highest dose of GR79236	**1)** GR79236 and GR190178 had a dose-dependent inhibitory effect on SSS stimulation-induced trigeminovascular activation in trigeminal nucleus caudalis (TNC) **2)** GR79236 and GR190178 inhibited SSS stimulation-induced CGRP release in cranial circulation **3)** DPCPX reversed the inhibitory effect of GR79236 on the trigeminal nucleus	Activation of adenosine A_1_ receptor causes neuronal inhibition without concurrent vasoconstriction, proposing a novel avenue for treating migraine and cluster headache
Haanes et al. [15]	To investigate the vasomotor effects of purinergic receptor in the middle meningeal artery (MMA), using functional myograph with natural and designed agonists	Rats (*n* = NR)	**1)** In vitro application of adenosine, caffeine and a A_2A_ receptor antagonist, SCH58261 **2)** mRNA expression was measured using PCR	**1)** Adenosine caused vasodilation in pre-contracted MMA segments **2)** Caffeine blocked adenosine-induced vasodilation and caused adenosine to elicit a contraction **3)** SCH58261 partly mimic the effect of caffeine **4)** All purinergic receptor mRNAs were present in the trigeminal ganglion at same base pair size as for MMA	Purinergic receptors might partly regulated blood flow through the MMA. Adenosine mainly binds to A_2A_ receptors, the strongest expressed adenosine receptor, to cause relaxation of MMAs. The relaxation is inhibited by SCH58261. Similar response is seen when adding physiological caffein concentrations (50 µM). This gives a putative molecular explanation for benefit and use of coffee/caffeine as a MMA vasoconstrictor potentially related to sensation of cranial pain
Haanes et al. [16]	To investigate the effects of five novel adenosine A_2A_ receptor antagonists on the vasodilation of the middle meningeal artery produced by an adenosine A_2_ receptor agonist (CGS21680) or endogenous CGRP	Rats (*n* = 57)	**1)** Intravenous adenosine and caffeine **2)** Periarterial electrical stimulation or intravenous CGS21680 followed by either i.v. bolus injections of JNJ-41942914, JNJ-39928122, JNJ-40529749, JNJ-40064440 and JNJ-41501798 Diameter was captured through closed cranial window	**1)** Adenosine caused dural arterial dilation and decreased blood pressure, which was inhibited by pre-treatment with caffeine **2)** Caffeine increased blood pressure **3)** JNJ antagonists did not affect electrical stimulated neurogenic vasodilation **4)** CGS21680 caused dural arterial dilation and decrease in blood pressure **5)** All JNJ-adenosine A_2A_ receptor antagonists blocked CGS21680-induced dural vasodilation with a more potent respond with A_2A_ over A_1_ selectivity	Selective A_2A_ receptor antagonists may offer a novel approach to antimigraine therapy that still needs to be determined
Hardebo et al. [17]	To investigate whether adenosine and closely related adenine compounds (AMP, cyclic AMP, ADP, and ATP) may cause a sufficiently high degree of vasodilatation in vitro to account for a possible involvement in initiating the vasodilatory phase of a migraine attack	Cats (*n* = 24) & Humans (*n* = 3)	In vitro application of adenosine, cAMP, ADP, and ATP to arteries (MCA, lingual and external maxillary artery), followed by measurement of tension and dilatory response	All adenine compounds caused **1)** dilation of pial arteries before and after application of prostaglandin F_2a_ (PGF_2a_) **2)** did not affect the extracranial arteries in both cats and humans **3)** a less pronounced dilation of pial arteries when extracellular K^+^ concentration was increased	Adenine compounds might initiate the dilatory phase in an attack or reactive hyperemia in intracranial circulation since a marked dilated of intracranial dilation was caused by these compounds
Honey et al. [19]	To investigate the effect of a selective adenosine A_1_ receptor agonist (GR79236) on neurogenic dural blood vessel dilation in anaesthetized rats	Rats (*n* = NR)	Electrically or CGRP evoked dural vasodilatation after treatment with an adenosine A_1_ receptor agonist, GR79236, or both GR79236 and an adenosine A_1_ receptor antagonist (DPCPX) through a cranial window	**1)** GR79236 dose-dependently inhibited electrically induced neurogenic vasodilation **2)** DPCPX reversed the inhibitory effect of GR79236 on electrically evoked vasodilation **3)** GR79236 did not inhibit CGRP induced vasodilation	It is possible that A_1_ agonists might be clinically effective in migraine because of an inhibitory effect both in the brain and periphery. This mechanism might offer a novel approach to migraine therapy
Jenkins et al. [22]	To investigate the receptors and mechanisms involved in prostanoid-induced CGRP release in cultured rat trigeminal neurons	Rats (*n* = NR)	In vitro application of adenosine deaminase to trigeminal neuronal culture	Adenosine deaminase did not alter baseline CGRP level nor CGRP release evoked by 1 µM PGE_2_	Not reported regarding adenosine
Lindquist et al. [31]	To investigate whether metabolic status could modulate adenosine accumulation in brain slices exposed to spreading depolarization (SD), and compare SD-associated adenosine release in vivo, under healthy, hypoglycemic, and ischemic conditions	Mice (*n* = NR)	Coronal slices were prepared at 250 μm, 350 μm, and 450 μm thicknesses. Adenosine measurements were done with amperometric recordings in brain slices in vivo	**1)** SD caused adenosine accumulation in vivo **2)** Adenosine signals triggered by SD could reliably report underlying metabolic status in brain slices	Adenosine or adenosine derivates might be useful as biomarkers of SD incidence in different clinical conditions
Lu et al. [32]	To investigate whether CGRP, A_2A_R and A_1_R are involved in migraine pain information transmission in the electrical stimulation of the trigeminal ganglion (ESTG) in migraine rat model and exploring the mechanisms of Tianshi capsule (TSC) as migraine treatment	Male rats (*n* = 40)	ESTG for 30 min. in one group, sham-operation without ESTG in another group and Tianshu capsule (TSC) followed by electrical stimulation of TG in the last group. The TNC and ipsilateral TG were removed for western blot analysis or RT-qPCR to evaluate CGRP, A_1_R and A_2A_R expression	**1)** Electrical stimulation increased CGRP and A_2A_R expression, and decreased A_1_R expression in the TNC and ipsilateral TG compared with the blank groups and sham-operated groups **2)** Treatment with TSC caused: - decreased CGRP and A_2A_R expression, - increased A_1_R expression in the TNC and ipsilateral TG compared to ESTG group	CGRP, A_1_R and A_2A_R mediates pain transmission and regulates the process during migraine. TSC regulates the expression of the three proteins
Wei et al. [46]	To investigate the possibility that nitric oxide donors, nitroglycerin and/or sodium nitroprusside activate trigeminovascular fibers by promoting neuropeptide release and vasodilation within the pial vasculature. Additionally, it was examined whether LY83583, a drug that lowers cyclic GMP, blocks the relaxation mediated by the topical application of the released neuropeptide CGRP or by sodium nitroprusside	Cats (*n* = 10)^a^	**1)** Application of adenosine before and after the CGRP antagonist (CGRP (8–37)) **2)** Application of adenosine before and after application of guanylate cyclase inhibitor (LY83583) Cranial window over the parietal cortex was used to observe arteries	**1)** CGRP-induced dilation was completely blocked by the CGRP antagonist, but the adenosine-induced vasodilation was not affected by the CGRP antagonist 2) Adenosine-induced vasodilation was not affected by guanylate cyclase inhibitor	Not reported regarding adenosine
Yegutkin et al. [48]	To investigate pro-nociceptive effects of adenine nucleotides in control and in migraine-like conditions modeled with the neuropeptide CGRP	Male rats (*n* = NR)	**1)** Bioluminescent and fluorometric techniques to measure purine levels in trigeminal ganglion cells, before and after pre-treatment with CGRP **2)** Electro-physiological recordings of nociceptive spikes in trigeminal nerves in meningeal tissues	**1)** Basal ATP and ADP levels in trigeminal cultures were maintained at very low level, meanwhile basal adenosine and AMP levels were almost one-two orders higher. CGRP pretreatment led to decreased adenosine levels by ∼ 50% in trigeminal cultures, but no changes in CGRP-treated meninges **2) **Adenosine could not activate nociceptive firing in meningeal nerves	Data are consistent with the purinergic hypothesis of migraine and proposes new targets against trigeminal pain

*A*_*1*_*R* A_1_ receptor, *A*_*2A*_*R* A_2A_ receptor, *ADP* adenosine 5’-diphosphate, *AMP* adenosine monophosphate, *ATP* adenosine triphosphate, *cAMP* cyclic adenosine 3’-5’-cyclic monophosphate, *CGRP* calcitonin gen-related peptide, *ESTG* electrical stimulation of the trigeminal ganglion, *MMA* meningeal media artery, *mRNA* messenger ribonucleic acid, *NR.* not reported, *PCR* polymerase chain reaction, *PGE*_*2*_ prostaglandin E2, *PGF*_*2a*_ prostaglandin F_2a_, *SD* spreading depressing, *SSS* superior sagittal sinus, *TG* trigeminal ganglion, *TNC* trigeminal nucleus caudalis, *TSC* Tianshu capsule, ^a^number of subjects exposed to adenosine related substances

### Narrative summaries

Arulmani et al. [1]. In pigs, intravenous infusion of adenosine A_1_ receptor agonist, GR79236, was compared to vehicle prior to capsaicin infusion. Total carotid blood flow, conductance, and plasma CGRP concentrations in jugular vein were assessed at baseline and after the infusions. GR79236 dose-dependently attenuated the capsaicin-induced carotid hemodynamic changes but not the CGRP release, compared to vehicle infusion.

Carruthers et al. [6]. In rats, adenosine agonists and antagonist (A_1_ receptor agonist GR79236X, adenosine A_1_ receptor antagonist, DPCPX, adenosine A_2A_ agonist, CGS21680, and A_3_ receptor agonist, 2-CI-IB-MECA) were applied to cultured trigeminal neurons in combination with forskolin or vehicle to induce release CGRP. Immunocytochemical studies and Western analysis assessed whether these pharmacological agents could modulate the forskolin induced CGRP release. GR79236X concentration-dependently inhibited forskolin-stimulated CGRP release, while DPCPX abolished GR79236X´s effect. CGS21680 and 2-CI-IB-MECA were unable to attenuate forskolin-induced CGRP secretion.

Faraci et al. [9]. Intravenous infusion of adenosine was administered to anesthetized dogs. Blood flow was measured with labelled, radioactive microspheres. Adenosine decreased aortic pressure along with blood flow and vascular resistance in the dura. Adenosine infusion did not alter cerebral blood flow.

Goadsby et al. [13]. Intravenous infusion of adenosine A_1_ receptor agonists were administered in anesthetized dogs, following electrical stimulation of the superior sagittal sinus (SSS). Jugular vein blood samples were taken at baseline, immediately after the SSS stimulation and following the A_1_ agonist infusion, for detection of CGRP levels. Both A_1_ receptor agonists, GR79236 and GR190178, inhibited SSS-induced activation in TNC and CGRP release in cranial circulation, in a dose-dependent manner. Moreover, adenosine A_1_ receptor antagonist, DPCPX, was able to reverse GR79236's inhibitory effect on TNC activation.

Haanes et al. [15]. Adenosine was applied to pre-contracted middle meningeal artery (MMA) segments isolated from rats. RT-PCR was used to characterize the expression of purinergic receptor and myography to access the vascular effects. Notably, all purinergic receptor mRNAs were detected in the trigeminal ganglion and MMA. Adenosine caused dilation of MMA, which was reversed by SCH58261 (A_2A_ receptor antagonist) and caffeine (adenosine receptor antagonist).

Haanes et al. [16]. Adenosine and caffeine were administered intravenously to six rats. Adenosine resulted in dural vasodilation and decrease in blood pressure. However, pre-treatment with caffeine inhibited adenosine´s effect. Caffeine caused an increase in blood pressure and a non-significant dilation of dural arteries. Secondly, intravenous infusions of different adenosine A_2A_ receptor antagonists (JNJ compounds) were given following intravenous administration of adenosine A_2A_ receptor agonist, CGS21680, or periarterial electrical stimulation (mode of CGRP-release), in rats. The closed cranial window was used to evaluate the antagonists´ effect on the CGS21680 and CGRP -induced dural dilation. CGS21680 caused vasodilation and decrease in arterial blood pressure. All A_2A_ receptor antagonists blocked CGS21680-induced dural vasodilation with a more potent respond with A_2A_ over A_1_ selectivity, while they did not affect electrical stimulated neurogenic vasodilation.

Hardebo et al. [17]. Adenosine, cAMP, ADP and ATP were applied in segments of middle cerebral artery and extracranial arteries of feline and humans. The dissected vessels were pre-constricted by prostaglandin F_2a_ (PGF_2a_) or 5-hydroxytryptamine (5-HT). The tension was measured with force displacement transducers and recorded on a Grass polygraph. All adenine compounds dilated feline pial arteries, however the dilatory response was less pronounced when extracellular K^+^ concentration increased. Adenine compounds did not influence the diameter of human and feline extracranial arteries.

Honey et al. [19]. In rats, intravenous infusion of adenosine A_1_-receptor agonist, GR79236, was compared to saline infusion in models of neurogenic dural vasodilation. Vasodilation was induced by either electrical stimulation of perivascular trigeminal nerves or intravenous CGRP. Cranial window was used to evaluate the vascular responses. GR79236 inhibited electrically induced neurogenic vasodilation in a dose-dependent manner but had no effect on vasodilation caused by CGRP. Selective A_1_ receptor antagonist, DPCPX, inhibited the effect of GR79236 on electrically evoked vasodilation, compared to vehicle.

Jenkins et al. [22]. Adenosine deaminase was applied in cultured rat trigeminal neurons. Application of prostaglandin E_2_ (PGE_2_) led to CGRP release from the cultured cells. Adenosine deaminase did not alter baseline or PGE2-evoked CGRP levels.

Lindquist et al. [31]. Accumulation of adenosine following spreading depolarization (SD) was investigated in brain slices of mice and in vivo. Amperometric recordings from adenosine-sensitive enzyme-linked electrochemical were made in brain slices and applied in vivo. SD generated transient adenosine accumulation in vivo which could reliably report underlying metabolic status in brain slices.

Lu et al. [32]. Εlectrical stimulation of the trigeminal ganglion (ESTG) or sham operation was performed in rats to investigate its effect on CGRP, adenosine A_1_ receptor and adenosine A_2A_ receptor expression. RT-qPCR and Western analysis was used for detection and quantification of the proteins. In the trigeminal nucleus caudalis (TNC) and ipsilateral trigeminal ganglion (TG), CGRP and A_2A_ expression increased following ESTG, while A_1_ decreased. Interestingly, pretreatment with hinese medicine Tianshu capsule (TSC) decreased CGRP and A_2A_ expression and increased A_1_ receptor expression.

Wei et al. [46]. In cats, the effect of topical application of adenosine and adenosine diphosphate was investigated before and following application of CGRP receptor antagonist, CGRP8-37. Cranial window was used to evaluate the vascular responses. CGRP8-37 was not able to reverse vasodilating effect of adenosine and adenosine diphosphate. Moreover, guanylate cyclase inhibitor, LY83583, had no effect on adenosine-induced vasodilation.

Yegutkin et al. [48]. The effects of adenine nucleotides were assessed in meninges of rats and cultured trigeminal cells following application of CGRP or placebo. Bioluminescent and fluorometric techniques were used to measure purine levels in trigeminal ganglion cells, and electrophysiology to record the nociceptive spikes in the meningeal trigeminal nerves, before and after pre-treatment with CGRP. CGRP decreased adenosine levels in cultured cells but not in the meninges, while adenosine was not able to activate nociceptive firing in the meningeal nerves. Moreover, basal levels of adenosine and AMP where higher compared to ATP and ADP in trigeminal cells.

## Discussion

The main findings of the present systematic review are that adenosine receptors modulate pain transmission through the TVS. While A_1_ receptor has an inhibitory effect, stimulation of A_2A_ receptor causes vasodilation and activation of trigeminal pain pathway.

In rats, electrical stimulation of the TG decreased adenosine A_1_ receptor expression and increased adenosine A_2A_ receptor expression in ipsilateral TG and TNC [32]. The former is suggested to be involved in migraine attack initiation, while upregulation of adenosine A_1_ receptors or activation of this receptor might block migraine attacks [32]. In support, one study found that treatment with adenosine A_1_ receptor agonists, GR79236 and GR190178, inhibited TVS activation after electrical stimulation of the superior sagittal sinus in cats [13]. Upregulation of both adenosine A_2A_ and CGRP receptors following electrical stimulation implies that when combined, the two receptors activate trigeminal pain transmission and cause migraine [32]. Overall, these findings implicate both adenosine A_1_ and A_2A_ receptors in pain regulation and transmission during migraine [13, 32].

Three studies showed that adenosine caused prominent dilation of pre-contracted middle meningeal artery, dural and pial arteries in vitro [15–17]. Another study showed that adenosine caused dural vasodilation in dogs [9]. Pretreatment with caffeine or adenosine A_2A_ receptor antagonist, SCH58261, was able to block adenosine-induced dilation in vitro [15, 16], suggesting that adenosine-induced vasodilation might be mainly dependent on adenosine A_2A_ receptor [15].

Adenosine A_1_ receptor agonist, GR79236, inhibited electrically-induced vasodilation and capsaicin-induced hemodynamic changes in carotid artery [1, 19]. Pretreatment with DPCPX prevented the inhibition following GR79236, indicating that its inhibitory or vasoconstricting effect is mediated through adenosine A_1_ receptor [19]. The same agonist, GR79236, inhibited CGRP release induced by adenylate cyclase activator, forskolin [6] without any effect on CGRP-induced vasodilation in rats [19]. These data indicate that GR79236 inhibits CGRP release via a pre-junctional inhibition, and that adenosine A_1_ receptors are present on CGRP-positive neurons [6, 13, 19]. Together with its vasoconstricting ability, it is suggested that GR79236 and adenosine A_1_ receptors hold anti-migraine potential [1, 6, 19].

In contrast to GR79236, adenosine A_2A_ receptor agonist, CGS21680, had no effect on forskolin-induced CGRP release [6]. However, CGS21680 caused dural vasodilation that was blocked by adenosine A_2A_ receptor antagonists (JNJ-compounds) [16]. The study showed that the lower the selectivity for A_2A_ receptor over A_1_ receptors, the higher the potential to attenuate the CGS21680 induced vasodilation. It was suggested that blocking both A_1_ and A_2A_ receptors might be necessary to completely attenuate dural vasodilation [16].

Of note, 2-CI-IB-MECA, an adenosine A_3_ receptor agonist, had no effect on forskolin-induced CGRP secretion in rats [6]. The involvement of adenosine A_3_ receptor in migraine has not been further investigated, however, adenosine A_3_ receptor agonist exhibited anti-nociceptive properties in models of chronic pain in rats and mice [24].

While CGRP antagonist, CGRP(8–37), and guanylate cyclase inhibitor, LY83583, inhibited CGRP and nitroglycerine induced vasodilation, both compounds did not alter adenosine-induced vasodilation [46]. This finding demonstrates that adenosine induced dilation is not dependent on activation of CGRP receptors or an increase in cyclic guanosine monophosphate. Another study showed that pretreatment of trigeminal ganglion cells with CGRP is followed by decreased adenosine levels compared to baseline [48]. It is suggested that the finding might be a part of migraine sensitization but due to other contradicting findings (i.e., no change in nociceptive firing), further investigation on adenosine’s mechanisms was recommended [48].

Collectively, studies showed adenosine receptors expression in the trigeminal pain pathway and indicated that adenosine-induced pronociceptive effect is mediated through A_2A_ receptor activation, whilst A_1_ receptor mediates antinociception. A_1_ receptor agonist, GR79236, inhibits activation of the TVS and vasodilation, while A_2A_ receptor antagonist, SCH58261, attenuated adenosine-induced vasodilation [13, 15, 19], designating adenosine A_1_ and A_2A_ receptors as possible targets in the treatment of migraine.

### Limitations and future perspective

The major limitations of the studies included, were differences in methodological approaches including designs, subjects, substances, and sampling sources. Additionally, concentrations and types of adenosine A_1_ receptor agonists applied, differed across the studies [6, 13, 19]. Different CGRP releasing mechanisms were applied throughout the studies, potentially affecting the potency of adenosine receptor agonists and antagonists in modulating the CGRP release [1, 6, 13, 22].

Human studies are needed to elucidate the headache inducing effect of adenosine in patients with migraine. A specific focus on adenosine A_1_ receptor agonists and A_2A_ receptors antagonists would be of great interest because of their potentially opposite effects based on current knowledge. Several adenosine receptor agonists and antagonist are currently available for research purpose only, while only one adenosine receptor antagonist, istradefylline, is currently U.S. Food and Drug Administration (FDA) approved as treatment for Parkinson’s disease (JF and RA 2020). To our best knowledge, no studies have been conducted on the adenosine A_2B_ receptor in migraine and adenosine A_3_ has only once been investigated in migraine [6]. This leaves a huge gap in our knowledge that needs to be explored in both clinical and pre-clinical setting.

## Conclusion

Preclinical data demonstrated that adenosine caused vasodilation and modulated CGRP release. We suggest that the adenosine A_1_ receptor and adenosine A_2A_ receptor could be potential targets for migraine treatment.

## Data Availability

Not applicable.

## Abbreviations

A_1_R: A_1_ receptor
A_2A_R: A_2A_ receptor
AC: Adenylyl cyclase
AMP: Adenosine monophosphate
ADP: Adenosine 5’-diphosphate
ATP: Adenosine triphosphate
cAMP: Cyclic adenosine monophosphate
CGRP: Calcitonin gene related peptide
DAG: Diacylglycerol
ES: Electrical stimulation
ESTG: Electrical stimulation of the trigeminal ganglion
FDA: U.S. Food and Drug Administration
GPCR: G-protein coupled receptors
ICHD: International Classification of Headache Disorders
IP_3_: Inositol 1,4,5-triphosphate
MAPK: Mitogen-activated protein kinase
MMA: Meningeal media artery
MO: Migraine without aura
mRNA: Messenger RNA
NR: Not reported
PKA: Protein kinase A
PCR: Polymerase chain reaction
PGE_2_: Prostaglandin E_2_
PGF_2a_: Prostaglandin F_2a_
PIP_2_: Phosphatidylinositol 4,5-biphosphate
PLC: Phospholipase C
PLD: Phospholipase D
SAH: S-adenosylhomocysteine
SD: Spreading depression
TG: Trigeminal ganglion
TNC: Trigeminal nucleus caudalis
TSC: Tianshu capsule
TVS: Trigeminovascular system
